# The role of recreational flow experience and well-being on re-participation intention: recreational sport participants

**DOI:** 10.3389/fpsyg.2025.1574337

**Published:** 2025-04-29

**Authors:** Alican Bayram, Ilimdar Yalcin, Enis Sahin, Nurullah Emir Ekinci, Laurentiu-Gabriel Talaghir, Teodora-Mihaela Iconomescu

**Affiliations:** ^1^Department of Recreation, Faculty of Sport Sciences, Bingol University, Bingol, Türkiye; ^2^Department of Coaching Education, Faculty of Sport Sciences, Bingol University, Bingol, Türkiye; ^3^Department of Recreation, Faculty of Sport Sciences, Yalova University, Yalova, Türkiye; ^4^Department of Individual Sports and Physical Therapy, Faculty of Physical Education and Sport, Dunarea de Jos University of Galati, Galați, Romania; ^5^Department of Sports Games and Physical Education, Faculty of Physical Education and Sport, Dunarea de Jos University of Galati, Galați, Romania

**Keywords:** sportive recreation, flow experience, well-being, behavioral intention, mental health

## Abstract

**Introduction:**

Recreational sports activities can offer participants many physical, psychological, and social benefits. The flow state they feel during participation can give them a sense of satisfaction and positively change their attitudes toward participation in recreational sports activities. This study aimed to determine the role of recreational flow experience and well-being on the re-participation intention in recreational sport participants.

**Method:**

A personal information form, the “Recreational Flow Experience Scale,” the “Recreational Sport Well-being Scale,” and the “Re-participation Intention Scale” were used as data collection tools. The sample group consisted of a total of 402 voluntary recreational sport participants including 191 women (47.5%) and 211 men (52.5%), who were selected by purposive sampling method in recreational parks and areas in Bingöl, Türkiye. The SPSS software was used to analyze data. Independent Samples *t*-test, One-Way ANOVA, Pearson Correlation analysis, and Regression analysis methods were used in the analysis process.

**Result:**

Statistically significant differences were observed between re-participation intention and the variables of gender, years of participation in sportive recreational activities, and frequency of participation. Statistically significant differences were also found between the frequency of participation in sportive recreational activities and life satisfaction and positive feelings subscales. There was also a statistically significant difference between the years of participation in sportive recreational activities and the recreational flow experience. A moderate positive correlation was found between recreational flow experience, recreational sport well-being, and re-participation intention variables.

**Discussion:**

Family flourishing and positive feelings have a significant relationship on individuals’ intention to re-participate in recreational activities. This suggests that emotional and social well-being may play an important role in the sustainability of participation in recreational sports. As the experience and frequency of participation in recreational sports activities increased, the levels of well-being elevated. Moreover, as the recreational flow experience and recreational well-being level elevated, the intention to re-participate in recreational sport activities increased. For this reason, organizing programs and studies to encourage continuous participation in recreational activities can benefit the well-being level of the society.

## Introduction

Flow theory describes a state in which individuals become completely focused on an activity, where their skill level and the challenge of the task are in balance. This process can enhance an individual’s motivation, alter their perception of time, and enable them to become fully immersed in the activity. The flow experience has been associated with optimal performance in areas such as sports, education, and work. Flow experience, as a psychological and performance-oriented concept, is one of the basic elements of positive psychology, which defines the moments when a person feels completely satisfied with an intense mood (Csikszentmihalyi, 1990). The flow experience indicates that the person is immersed or engaged in an activity that distracts from unrelated emotions and thoughts (Perttula et al., 2017; Turan, 2019). This experiential process is a subjective psychological state in which an individual becomes deeply immersed in an activity (Fullagar et al., 2013). During this experience, the perception of time and surroundings fades, and a high level of motivation to engage in the activity is achieved (Ottiger et al., 2021; Abuhamdeh, 2020). Attention is fully directed toward the activity, and the individual becomes completely absorbed in it (Marty-Dugas et al., 2021). During the flow experience process, participants have an optimal experience between skill and difficulty (Mao et al., 2016). Ensuring balance prevents them from experiencing boredom, stress or anxiety. The optimal experience allows those who are distracted from the problems to focus on the activity. This optimal experience keeps them in the flow channel. The balance of skill and difficulty has been theorized to show the signs of flow (Moneta, 2004). People can encounter difficulties in cases where the skill is insufficient to maintain the activity (Kiili et al., 2012). In this process, they may experience negative emotions such as stress, anxiety and fear. If there is a balance between skill and difficulty in the activity, individuals are satisfied and experiences the flow process with pleasure (Orhun and Gülcan, 2022; Keller and Blomann, 2008). The flow process continues when they persist an activity with a high level of interest, concentration and pleasure. In this process, they usually experience a loss of intrinsic interest and time perception and experience a high degree of happiness (Rha et al., 2005; Voiskounsky et al., 2004; Ayhan and Eskiler, 2024). This feeling of happiness can help them to continue the activity and elevate their level of skill and difficulty. At this point, happiness is not merely the result of luck or fortune but is experienced through the individual’s engagement in an activity, fostering internal satisfaction and a sense of control over their experiences. In this way, the individual achieves an optimal experience or a state of flow (Csikszentmihalyi and LeFevre, 1989; Csikszentmihalyi and Hunter, 2003; Collins et al., 2009).

In the flow experience, the balanced interaction between the difficulty of the task and the individual’s skill level enables the person to achieve a sense of satisfaction (Zhang and Abd Rahman, 2022). The optimal experience, sense of satisfaction, and state of flow achieved through participation in an activity can influence behavioral intentions (Ding and Hung, 2021; Uhm et al., 2022). Participants may want to participate again in any activity in which they feel a sense of satisfaction. Within the framework of the theory of planned behavior, an individual’s prior experience with a behavior or activity, coupled with a strong motivation toward that behavior, can trigger the intention to participate in the activity again (Ajzen, 1991; Karakullukcu et al., 2025). This re-participation is determined by behavioral intention. Behavioral intention includes positive and negative individual judgments toward the actions that people may perform in the future (Ma and Kaplanidou, 2021; Avan and Karaküçük, 2024). In behavioral intention, the activity participants want to re-participate in an activity that they are satisfied with and recommend it to others (Li et al., 2020; Demirhan and Eskiler, 2018). At this point, behavioral intention shows their willingness to perform an action (Filo et al., 2013). Thus, one’s intention to re-participate appears as the state of exhibiting behavior again for an action that they have previously experienced depending on the level of motivation (Ayhan, 2022). The individual’s intention to participate again, along with their actual engagement in the activity, may also influence the re-experiencing of the flow state (Kim, 2022). Flow experience is a situation that can be experienced not only in a person’s work or compulsory activities, but also in leisure time activities that can provide recreational participation.

With the flow experience, the desire to re-participation in an activity can sometimes be a compulsory work or a social activity that the person participates in for recreational purposes. These recreational activities include spending time in a park, engaging in art, participating in sports organizations, having a hobby, participating in metaleisure activities, or sometimes doing nothing (Reed et al., 2025), and metaleisure or metarec activities in 3 or more dimensional universes (Bayram, 2022). In the case of sporting, artistic or casual participation for recreational purposes, it can be considered that people can make use of the benefits of this type of participation at a high level. Among these, recreational sports activities include the evaluation of sportive activities for leisure and entertainment purposes depending on various physical activities (Ayar, 2017; Lower et al., 2013). At this point, recreational sports activities can be considered as non-occupational, voluntary and leisure physical activities and exercises (Durhan and Karaküçük, 2020; Yalçın and Ayhan, 2020). It is an area where less physical and mental effort is exhibited compared to sports, which are based on competition and struggle, aiming at the improvement of various elements such as entertainment, socialization, health and physical fitness (Kenefick and Cheuvront, 2012).

Participation in recreational sports activities can significantly increase the psychological and physiological well-being of individuals and this may increase their happiness in direct proportion. Previous studies reported that if people participate in recreational sports activities, they physically and psychologically feel better and experience positive emotions (Chatzisarantis and Hagger, 2007), their stress decreases, their general mood improves and their depressive and anxiety symptoms are alleviated (Penedo and Dahn, 2005). When examining from physiological aspect, it is seen that recreational activities improve muscle strength, cardiovascular health, and physical fitness (Warburton et al., 2006). Participants who feel well-being with recreational sports feel positive emotions and achieve success (Gümüşay et al., 2023). Recreational sport well-being refers to the state of well-being offered by recreational sporting activities (Koç, 2022). Recreational flow experience enhances psychological well-being and satisfaction, fostering positive emotions and engagement in activities (Cho and Shin, 2020; Decloe et al., 2009; Ayhan et al., 2020). During the process of recreational flow experience, participants can experience positive emotions such as joy, happiness and a sense of achievement. These positive emotions enable them to participate in activities again (Wöran and Arnberger, 2012). With the flow experience experienced in participation in sportive recreational activities, the person shows a willingness to re-participate in the activity with the perceived benefit and interest in the activity (Ayhan, 2022). The psychological benefits provided by activities can lead to improvements in individuals’ problem-solving skills and self-esteem. Recreational sports activities contribute positively to the physical and psychological health of individuals and are important for society. Participation in such activities leads to an increase in individuals’ overall quality of life and life satisfaction (Al Ahmed, 2024; Mutz et al., 2021; Stenseng et al., 2015). However, participants’ sustainability in participation in sportive activities depends on various factors. Among these factors, the flow experience and well-being that participants feel during the sportive activity process are essential. In this context, this study aimed to examine the effect of flow experience and well-being experienced by recreational sports participants on their intention to re-participate in activities. Examining the effect of flow experience and well-being on the re-participation intention will provide important information about long-term participation in recreational sports activities. Numerous studies have investigated the concepts of recreational flow experience, recreational well-being and re-participation intention, which can affect human psychology with participation in recreational sports (Gumus et al., 2021; Ayhan and Alanoglu, 2023; Akçakese et al., 2024). In the study, the relationship between recreational flow experience, recreational well-being, and re-participation intention, as well as the effects among various variables, were examined. Considering this information, the significance of this study emerges to explain the well-being and re-participation intention among recreational sports participants as a result of experiencing flow. At this point, we focused on the question of how recreational flow experience and well-being affect recreational sport participants’ re-participation intention. In this sense, the results of this study will contribute to literature. In addition, the results of the study aim to explain the effect of flow experience on physical and mental health, life satisfaction, family and social relationships, general well-being and happiness in life in those who participate in recreational sports activities. For this reason, the following hypotheses were established.

H1: There is a positive relationship between Recreational flow experience, recreational well-being and re-participation intention.

H2: As recreational flow experience and recreational well-being increase, re-participation intention also increases.

## Methods

### Research model and participants

The study design is cross-sectional and exploratory of relationships. The research group consists of 402 individuals selected through purposive sampling from individuals who voluntarily participated in recreational sports activities in parks and recreation areas in Bingöl, Türkiye. In determining the sample size, the unlimited population sampling formula (*n* = p.q.z 2 *α*/e2) was used. The sample size was determined as 384 people (*n* = p.q.z 2 α/e2 = 1.962 × 0.5 × 0.5 / 0.052 = 3.8416 × 0.25 / 0.0025 = 384) at 5% significance level and 5% sampling error by taking into account the ratio that maximizes the variance (p: 0.50) (Ural and Kılıç, 2006). Within the scope of the research, data were collected from 450 volunteer individuals. 48 incomplete or inaccurate questionnaires were excluded from the scope of the research and 402 individuals were included in the scope of the research. A total of 402 individuals engaged in recreational sports including 211 men (52.5%) and 191 women (47.5%) participated in the study (Table 1). The data were collected through face-to-face interviews with recreational sports participants between 15 May 2024 and 15 June 2024, using structured questionnaires and scales to gather relevant data.

**Table 1:** Descriptive results of participants’ demographic variables.

Variables	Category	*n*	%
Age	18–20 years	96	23.9
	21–23 years	188	46.8
	24 years and older	118	29.3
Gender	Female	191	47.5
	Male	211	52.5
Monthly income (Turkish Lira)	0–5,000	265	65.9
	5,001–10,000	48	11.9
	10,001–15,000	31	7.7
	15,001 and above	58	14.5
Years of participation in sportive recreational activities	1–3 years	127	31.6
	3–5 years	61	15.2
	5–7 years	97	24.1
	7 years and above	117	29.1
Frequency of participation in sportive recreational activities	1–2 days a week	227	56.5
	3–4 days a week	121	30.1
	5–7 days a week	54	13.4

### Data collection tools

In addition to a personal information form used to determine the demographic characteristics of the participants, the “Recreational Flow Experience Scale,” the “Recreational Sport Well-being Scale,” and the “Re-participation Intention Scale” were used.

#### Personal information form

In order to determine the personal characteristics of the research participants, questions on age, gender, monthly income, years of participation in sportive recreational activities, and frequency of participation in sportive recreational activities were included.

#### Recreational flow experience scale

The Recreational Flow Experience Scale, developed by Ayhan et al. (2020), was used to determine the flow states perceived by the participants during the recreational activity. The scale consists of 9 items that are rated using a 7-point Likert scale [(1) Strongly disagree, …, (7) Strongly agree] with a single dimension. There are no reverse-coded items in the scale, and the minimum score is 1 while the maximum score is 7. According to the validity and reliability statistical results for the recreational flow experience scale, it was determined that Cronbach’s *α* coefficient was *α* = 0.94. Additionally, the test–retest analysis indicated that the inter-item correlation coefficient *r* = 0.81. In the present study, Cronbach’s α coefficient for the recreational flow experience scale was found to be *α* = 0.90. A high mean value obtained from the scale indicates that the recreational flow experience is strongly experienced, while a low mean value suggests that the recreational flow experience is weakly experienced.

#### Recreational sport well-being scale

The Recreational Sport Well-Being Scale, developed by Pi et al. (2022) and adapted to Turkish by Koç (2022), was used to assess the well-being obtained by the participants from their recreational sports activity experiences. The scale has 14 items and 4 subscales (physical and mental health, life satisfaction, family flourishing, and positive feelings). The items of the scale are rated using a 5-point likert scale [(1) Strongly disagree, …, (5) Strongly agree]. There are no reverse-coded items in the scale, and the minimum score is 1 while the maximum score is 5. Cronbach’s *α* coefficients of the subscales of the scale were found as α = 0.83 for physical and mental health, *α* = 0.80 for life satisfaction, *α* = 0.88 for family flourishing, and α = 0.81 for positive feelings. In the present study, they were found to be *α* = 0.68, *α* = 0.78, *α* = 0.89, and *α* = 0.85, respectively.

#### Re-participation intention scale

The Re-Participation Intention Scale, developed by Şimşek (2011) was used to determine the participants’ re-participation intention in recreational activities. The scale consists of 6 items and one dimension. The items of the scale are scored on a 5-point Likert scale [(1) Strongly disagree, …, (5) Strongly agree] and 2 items are reverse coded. There are no reverse-coded items in the scale, and the minimum score is 1 while the maximum score is 5. In the validity and reliability analysis of the scale, Cronbach’s α coefficient was *α* = 0.69 and composite reliability value was CR = 0.74. In the present study, the Cronbach’s α coefficient of the scale was determined as *α* = 0.77. The high mean scores signify that the re-participate intention is high.

### Ethical considerations

This study was found to be ethically appropriate upon the decision of the Bingöl University Health Sciences Scientific Research and Publication Ethics Committee numbered E-33117789-770-157854.

### Statistical analysis

The data of the study was analyzed through the SPSS statistical software. Descriptive statistics (percentage, frequency, mean, and standard deviation) were used to determine the demographic characteristics of the participants. Whether or not the data were normally distributed was determined based on skewness and kurtosis values (Table 2). Parametric tests were used since the data were normally distributed. One-way ANOVA test, Independent *t*-test, Pearson Correlation analysis, and Linear Regression analysis were used for normally distributed data. *Post hoc* (Tukey) analysis was used to determine the difference between the groups in the ANOVA test. Significance was set at *p* < 0.01 and *p* < 0.05.

**Table 2:** Skewness and kurtosis values for recreational flow experience, recreational sport well-being, and re-participation intention scales.

Variables	*N*	Minimum	Maximum	Mean	Skewness	Kurtosis
Recreational flow experience	402	3.00	7.00	6.04	−0.672	1.065
Physical and mental health	402	2.50	5.00	4.08	0.053	−0.052
Life satisfaction	402	2.75	5.00	4.25	−0.165	−0.404
Family flourishing	402	1.00	5.00	3.82	−0.638	,586
Positive feelings	402	2.00	5.00	4.28	−0.209	−0.186
Re-participation intention	402	2.00	5.00	4.18	−0.447	0.576

When Table 2 is examined, it is determined that the Skewness and Kurtosis values of the recreational flow experience scale, recreational sport well-being scale and re-participation intention scale are between −1.5 and +1.5 points. Tabachnick et al. (2007) emphasized that Skewness and Kurtosis values between −1.5 and +1.5 points show normal distribution of the data and parametric tests should be used.

## Results

When Table 3 was analyzed, a statistically significant difference was found between the gender variable and the re-participation intention (*p* < 0.05). This difference indicates that male individuals have a higher re-participation intention than female individuals. No statistically significant difference was found between the recreational flow experience scale, and physical and mental health, life satisfaction, family flourishing, and positive feelings subscales of recreational sport well-being scale according to gender variable (*p* > 0.05).

**Table 3:** Construct *t*-test results regarding recreational flow experience scale, re-participation intention scale, and subscales of recreational sport well-being scale according to participants’ gender.

Scales	Gender	*N*	Mean	SD	*t*	*p*
Recreational flow experience	Female	191	6.05	0.66	0.233	0.816
	Male	211	6.03	0.69		
Physical and mental health	Female	191	4.09	0.49	0.575	0.566
	Male	211	4.06	0.51		
Life satisfaction	Female	191	4.23	0.56	0.542	0.588
	Male	211	4.26	0.52		
Family flourishing	Female	191	3.78	0.79	0.998	0.319
	Male	211	3.86	0.83		
Positive feelings	Female	191	4.26	0.54	0.790	0.430
	Male	211	4.30	0.57		
Re-participation intention	Female	191	4.12	0.56	−1.976	0.049*
	Male	211	4.23	0.56		

*p < 0.05.

When Table 4 was examined, it was observed that there was a statistically significant difference between the years of participation in sportive recreational activities and the recreational flow experience scale, re-participation intention scale, and recreational sport well-being scale’s life satisfaction, family flourishing, and positive feelings subscales (*p* < 0.05). It has been found that as the number of years of participation in sportive recreational activities increases, the recreational flow experience, recreational well-being and re-participation intention also increase. No statistically significant difference was detected between the physical and mental health subscale and participants’ years of participation in sportive recreational activities (*p* > 0.05).

**Table 4:** ANOVA results regarding recreational flow experience scale, re-participation intention scale, and subscales of recreational sport well-being scale according to participants’ years of participation in sportive recreational activities.

Scales	Years of participation in sportive recreational activities	*N*	Mean	SD	*F*	*p*	*Post hoc*
Recreational flow experience	1–3 years^A^	127	5.79	0.67	9.643	0.000*	A-B,C,D
	3–5 years^B^	61	6.08	0.59			
	5–7 years^C^	97	6.13	0.73			
	7 years and above^D^	117	6.22	0.60			
Physical and mental health	1–3 years^A^	127	4.00	0.49	0.503	0.213	-
	3–5 years^B^	61	4.10	0.52			
	5–7 years^C^	97	4.12	0.54			
	7 years and above^D^	117	4.11	0.46			
Life satisfaction	1–3 years^A^	127	4.09	0.54	5.361	0.001*	A-B,C,D
	3–5 years^B^	61	4.34	0.55			
	5–7 years^C^	97	4.35	0.53			
	7 years and above^D^	117	4.28	0.50			
Family flourishing	1–3 years^A^	127	3.67	0.78	3.041	0.029*	A-C
	3-5 years^B^	61	3.89	0.82			
	5–7 years^C^	97	3.99	0.82			
	7 years and above^D^	117	3.81	0.82			
Positive feelings	1–3 years^A^	127	4.18	0.52	4.141	0.007*	A-C
	3-5 years^B^	61	4.31	0.50			
	5–7 years^C^	97	4.43	0.57			
	7 years and above^D^	117	4.25	0.58			
Re-participation intention	1–3 years^A^	127	3.98	0.58	8.739	0.000*	A-B,C,D
	3–5 years^B^	61	4.25	0.55			
	5–7 years^C^	97	4.24	0.55			
	7 years and above^D^	117	4.31	0.50			

*p < 0.05; *Post hoc* (Tukey). A–D represents groups and - represents differences between groups.

When Table 5 was examined, a statistically significant difference was found between the frequency of participation in sportive recreational activities and life satisfaction and positive feelings subscales and re-participation intention scale (*p* < 0.05). This difference is between those who participate 1–2 times a week and those who participate 3–4 times a week in life satisfaction and positive feelings subscales and it is in favor of those who participate 3–4 times a week. In addition, it was found that as the frequency of weekly participation in recreational sport activities increased, re-participation intention also increased. No statistically significant difference was found between the recreational flow experience scale, family flourishing and physical and mental health subscales and the frequency of participation in sportive recreational activities (*p* > 0.05).

**Table 5:** ANOVA results regarding recreational flow experience scale, re-participation intention scale, and subscales of recreational sport well-being scale according to participants’ frequency of participation in sportive recreational activities.

Scales	Frequency of participation in activities	*N*	Mean	SD	*F*	*p*	Post hoc
Recreational flow experience	1–2 days a week^A^	227	5.93	0.66	9.061	0.267	-
	3–4 days a week^B^	121	6.14	0.64			
	5–7 days a week^C^	54	6.30	0.73			
Physical and mental health	1–2 days a week^A^	227	4.04	0.49	1.325	0.267	-
	3–4 days a week^B^	121	4.12	0.50			
	5–7 days a week^C^	54	4.12	0.54			
Life satisfaction	1–2 days a week^A^	227	4.17	0.52	5.025	0.007*	A-B
	3-4 days a week^B^	121	4.35	0.55			
	5–7 days a week^C^	54	4.33	0.52			
Family flourishing	1–2 days a week^A^	227	3.75	0.77	1.812	0.165	-
	3–4 days a week^B^	121	3.91	0.86			
	5–7 days a week^C^	54	3.89	0.88			
Positive feelings	1–2 days a week^A^	227	4.21	0.56	4.890	0.008*	A-B
	3-4 days a week^B^	121	4.40	0.50			
	5–7 days a week^C^	54	4.31	0.61			
Re-participation intention	1–2 days a week^A^	227	4.08	0.57	9.554	0.000*	A-B,C
	3-4 days a week^B^	121	4.26	0.50			
	5–7 days a week^C^	54	4.40	0.58			

*p < 0.05; *Post hoc* (Tukey). A–C represents groups and - represents differences between groups.

When the results of the analysis in Table 6 were analyzed, it was determined that there was a positive and moderately significant correlation between the recreational flow experience scale, subscales (physical and mental health, life satisfaction, family flourishing, positive feelings) of recreational sport well-being scale, and the re-participation intention scale (*p* < 0.05).

**Table 6:** Pearson correlation analysis results regarding recreational flow experience scale, re-participation intention scale, and subscales of recreational sport well-being scale.

Variables		(1)	(2)	(3)	(4)	(5)	(6)
Recreational flow experience (1)	*r*	1					
	*p*						
Physical and mental health (2)	*r*	0.391**	1				
	*p*	0.000					
Life satisfaction (3)	*r*	0.419**	0.589**	1			
	*p*	0.000	0.000				
Family flourishing (4)	*r*	0.316**	0.380**	0.389**	1		
	*p*	0.000	0.000	0.000			
Positive feelings (5)	*r*	0.464**	0.474**	0.573**	0.365**	1	
	*p*	0.000	0.000	0.000	0.000		
Re-participation intention (6)	*r*	0.483**	0.317**	0.386**	0.341**	0.417**	1
	*p*	0.000	0.000	0.000	0.000	0.000	

*N*: 402; **p < 0.01.

When Table 7 is examined, multiple regression analysis was used to determine the effect of recreational flow experience on re-participation intention. According to the results of the analysis, it was determined that the recreational flow experience variable had a significant positive effect on the re-participation intention (*B* = 0.483, *p* < 0.01). Based on this finding, the flow experience felt as a result of participation in recreational sports activities can also increase the re-participation intention. As a result of the analysis, it was determined that the recreational flow experience predicted the re-participation intention by 23% (_Adj._R^2^ = 0.231).

**Table 7:** The effect of recreational flow experience scale on re-participation intention.

Dependent variable: re-participation intention						
Variables	*B*	*t*	*p*	*R* ^2^	_Adj._R^2^	*F*
Constant		7.965	0.001	0.233	0.231	121.702
Recreational flow experience	0.483	11.032	0.001			

*p < 0.01.*

When Table 8 is examined, multiple regression analysis was used to determine the effect of recreational sport well-being sub-dimensions on the re-participation intention. According to the results of the analysis, it was determined that the family flourishing development sub-dimension variable had a significant positive effect on the re-participation intention (*B* = 0.175, *p* < 0.01). In addition, the positive feelings sub-dimension variable was found to have a significant positive effect on the re-participation intention (*B* = 0.246, *p* < 0.01). Based on this finding, recreational sports can increase re-participation intention by increasing well-being in family flourishing and positive feelings. In addition, it was determined that the physical and mental health and life satisfaction sub-dimensions variables did not have an effect on the re-participation intention. As a result of the analysis, it was determined that recreational sport well-being sub-dimensions predicted the re-participation intention by 22% (_Adj._R^2^ = 0.227).

**Table 8:** The effect of recreational sport well-being sub-dimensions on re-participation intention.

Dependent variable: re-participation intention						
Variables	*B*	*t*	*p*	*R* ^2^	_Adj._R^2^	*F*
Constant		7.519	0.001	0.235	0.227	30.450
Physical and mental health	0.045	0.802	0.423			
Life satisfaction	0.151	2.500	0.013			
Family flourishing	0.175	3.556	0.001			
Positive feelings	0.246	4.434	0.001			

p < 0.01.

## Discussion

The findings of the present study, which was conducted to examine the role of recreational flow experience and well-being on re-participation intention in recreational sports participants and to determine whether there are differences between the variables, were interpreted.

The analysis made based on gender variable indicated no significant difference in terms of recreational flow and well-being. Studies indicate that there is no significant difference in flow experience based on gender, and this finding is supported by research in the literature (Stavrou et al., 2007; Korer and Alpullu, 2020; Mouelhi-Guizani et al., 2023; Yapıcı et al., 2022; Bedir, 2023). Additionally, well-being does not appear to be influenced by gender (Joshi, 2010; Wolsko et al., 2019; Köksal et al., 2023; Gümüşay et al., 2023; Koç et al., 2024). Similarly, no relationship has been found between gender and the fulfillment of psychological needs (Gündoğdu and Yavuzer, 2012), happiness (Mehrdadi et al., 2016), or recreational benefits (Sahin and Yalcin, 2024). However, regardless of gender the high re-participation intention indicated that individuals’ desire to re-participate in recreational activities increased with the psychological benefits they obtained from these activities. These results were consistent with the studies reporting that recreational activities support not only physical health but also psychosocial well-being including life satisfaction, social adaptation, and strengthening of social support networks (Penedo and Dahn, 2005; Warburton et al., 2006; Alanoglu et al., 2020). In addition, such activities strengthen individuals’ ability to cope with stress and deepen their social relationships (Cohen and Wills, 1985; Keller and Blomann, 2008; Carbonneau et al., 2015; Beneton et al., 2017).

In the study, significant differences were found in the recreational flow and well-being according to the years of participation in recreational sports activities, except for the physical and mental health subscale. With long years of participation in sportive recreational activities, there is an increase in the re-participation intention due to the increase in flow level, life satisfaction, family flourishing and positive feelings. This situation is compatible with the results in the studies of Patten et al. (2013), Demirhan and Eskiler (2018) and Koç (2022) reporting that recreational activities enhance the quality of life of participants by providing social harmony and social commitment to them, rather than just providing physical benefits. In addition, continuous participation in recreational activities increases recovery (Tasiemski et al., 2006), in particular, long-term participation in sportive recreational activities develops healthy living habits and increases positive feelings (Spivey and Hritz, 2013). In addition, it is seen that the state of flow felt through participation in long-term recreational activities makes people satisfied with their lives (Chang, 2016). In addition, the state of flow has the potential to improve social relations (Heo et al., 2010). Recreational sports increase individuals’ happiness levels by being active in their social lives and improving their social characteristics (Kurniawan et al., 2022). In this context, it can be asserted that individuals intend not only to be healthy but also to strengthen their social ties and maintain a life in social harmony. In addition, continuous participation in recreational activities is decisive in experiencing a state of flow and developing social competence (Duman et al., 2022). Continuous participation in sportive recreational activities is an important factor in experiencing positive feelings such as a sense of flow, pleasure and satisfaction, and emotional relaxation such as reduced stress and anxiety (Decloe et al., 2009). Although the difficulty of the activity is a stressful factor in the flow experience, stress decreases with the experience of the flow state and happiness appears as a reflection of positive mood (Hamilton et al., 2019; Peifer et al., 2014).

Results showing that the frequency of participation in sports recreational activities was associated with the recreational flow, recreational well-being, and re-participation intentions of the participants also demonstrated that individuals who participated frequently preferred these activities primarily for the improvement of their life satisfaction and positive emotional states. This suggest that individuals consider recreational activities not only as a goal but also to maintain a healthy and happy life. In addition, it was found that the frequency of participation in recreational activities emphasized psychological benefits in participants rather than physical health. These results are compatible with studies highlighting those recreational activities improved individuals’ life satisfaction, strengthened their social ties, and contributed to social welfare (Solish et al., 2010; Wöran and Arnberger, 2012; Litwiller et al., 2017; Keskin and Bayram, 2018; Tian et al., 2022). Especially in participation in adventure recreational sports activities, the feeling of flow contributes to a high degree of happiness, self-discovery and continuity in the motivation to participate in the activity (Boudreau et al., 2020; Jackson et al., 2023). Participation in fitness activities for recreational purposes can also lead to re-participation in the activity by experiencing a sense of flow and satisfaction (Jeon et al., 2021; Doğan and Ünal, 2024).

The results (Tables 6–8) support the main motivation of the study, which confirmed the predictions of flow theory and recreational well-being theory. The main motivation of the study was to explain individuals’ re-participation intentions in recreational activities by associating the principles of Csikszentmihalyi's (1990) flow theory with the principles of recreational well-being theory developed by Pi et al. (2022) and adapted to Turkish by Koç (2022). Correlation and regression analyses showed that the enjoyment and well-being of individuals participating in recreational activities were positively associated with re-participation. This is parallel with previous studies suggesting that high intrinsic motivation and a sense of satisfaction encourage individuals to continue participating in activities (Kiili et al., 2012; Mao et al., 2016; Perttula et al., 2017; Ding and Hung, 2021; Kim, 2022; Uhm et al., 2022; Karakullukcu et al., 2025). Additionally, these results are consistent with other studies demonstrating that flow experience is significantly associated with well-being (Carpentier et al., 2012; Cheng and Lu, 2015; Moutinho et al., 2019; Tse et al., 2021; Ayhan and Alanoglu, 2023) and happiness (Collins et al., 2009; Sahoo and Sahu, 2009; Tsaur et al., 2013; Cho and Shin, 2020; Ayhan and Eskiler, 2024).

In the study, it was observed that recreational flow and well-being experiences strengthened the participants’ life satisfaction, family flourishing and positive feelings, thus leading to increase their motivation to participate in recreational sports activities. These results are also consistent with social support theory. According to the social support theory of Cohen and Wills (1985), social interactions improve the psychological resilience of individuals and enhance their quality of life. In particular, the flow experience, when a person fully immerses himself in an activity, provides high satisfaction and continuity of participation in these individuals, and is valuable in terms of strengthening social harmony and social ties (Keller and Blomann, 2008; Turan, 2019). The flow state, which reinforces positive emotions, has the potential to increase recreational benefit, motivation to participate in recreational activities, sense of freedom and intrinsic motivation (Hsu and Liu, 2020; Bum et al., 2022; Jackson et al., 2023).

## Conclusion

This study contributes to the literature by evaluating the interaction of flow theory and recreational well-being with re-participation intention in a relational framework. The findings of this study demonstrate that flow experience and recreational well-being significantly enhance re-participation intentions among recreational sports participants. These results corroborate Hypotheses 1 and 2, confirming that recreational flow experience and well-being positively correlation re-participation intention (Table 9). Flow experience heightened intrinsic motivation, fostering greater enjoyment and satisfaction, thereby reinforcing participants’ propensity for future re-participation. Morever, recreational sport activities increased participants’ physical and psychological well-being and improved life satisfaction. The strengthening of family relationships and the increase in positive emotions in sportive recreational activity participation create an effect that supports the general welfare of the society. At this point, recreational sports have a positive relationship with individuals’ social cohesion and social welfare in addition to their physical and mental health. Long-term participation in recreational sports activities has a positive effect on recreational flow experience and re-participation intention. This situation contributes to the formation of individuals who practice recreational sports activities as a lifestyle, have high levels of well-being and recreational flow, and are continuous activity participants.

**Table 9:** Hypothesis results.

Hypothesis	Results
H1: There is a positive relationship between recreational flow experience, recreational well-being and re-participation intention.	Supported
H2: As recreational flow experience and recreational well-being increase, re-participation intention also increases.	Supported

### Suggestions

Given the lower re-participation intention among female participants, policymakers and practitioners should diversify activity offerings and enhance accessibility to encourage women’s sustained engagement.

In the first years of participation in recreational sports, the level of recreational flow, recreational sport well-being and re-participation intention were observed to be low. It is recommended that activity practitioners should receive professional trainings from experts to ensure the highest efficiency from the activities.

Social well-being and happiness can be promoted by service providers, experts and relevant departments by facilitating community participation in activities and making recreational sports a way of life for the community.

Considering that recreational flow experience, well-being and re-participation intention increase as the frequency of participation in sportive recreational activities increases, it is recommended that education and support services should be provided to all segments of the society in order to make recreational sports a lifestyle of the society.

Considering the potential of recreational sports to increase positive emotions and social relations, organizing recreational sports organizations and ensuring the participation of individuals from all aspects of society reveals the potential to increase the number of social well-being and happy individuals.

### Limitations

There are some limitations that should be considered when interpreting the results of this study. The study’s reliance on participants from a single geographic region and specific recreational settings may limit the generalizability of the results. Participants who actively practiced sportive recreation activities participated in the study. The absence of passive or disadvantaged recreational participants may limit the results of the study. In the study, data were collected through purposive sampling. Since this method usually focuses on a specific group selected according to the research purpose, it limits the generalizability of the results obtained to the whole population of sportive recreational activities. Furthermore, the correlational design of the study limits the ability to draw causal conclusions. Longitudinal studies are needed to better understand the causal relationships between flow experience, well-being, and re-participation intention.

## Data Availability

The raw data supporting the conclusions of this article will be made available by the authors, without undue reservation.
